# Plant community assembly is jointly shaped by environmental and dispersal filtering along elevation gradients in a semiarid area, China

**DOI:** 10.3389/fpls.2022.1041742

**Published:** 2022-11-25

**Authors:** Jie Zheng, Muhammad Arif, Xinrui He, Dongdong Ding, Songlin Zhang, Xilu Ni, Changxiao Li

**Affiliations:** ^1^ Key Laboratory of Eco-Environments in the Three Gorges Reservoir Region (Ministry of Education), Chongqing Key Laboratory of Plant Ecology and Resources Research in the Three Gorges Reservoir Region, School of Life Sciences, Southwest University, Chongqing, China; ^2^ Biological Science Research Center, Academy for Advanced Interdisciplinary Studies, Southwest University, Chongqing, China; ^3^ State Key Laboratory of Seedling Bioengineering, Ningxia Forestry Institute, Yinchuan, China

**Keywords:** dryland, community assembly, taxonomic diversity, phylogenetic diversity, Helan Mountain Nature Reserve

## Abstract

Environmental filtering (EF) and dispersal filtering (DF) are widely known to shape plant community assembly. Particularly in arid and semi-arid mountainous regions, however, it remains unclear whether EF or DF dominate in the community assembly of different life forms or how they interact along elevational gradients. This research aims to reveal how different ecological processes influence herbaceous and woody community assembly and how they respond to various environmental drivers and elevational gradients. Here we integrated taxonomic diversity (TD), phylogenetic diversity (PD), and ecological drivers across an elevational gradient of 1,420 m in the Helan Mountain Nature Reserve, in typical arid and semi-arid areas of China. This study showed that the TD and PD of herbaceous communities significantly increase linearly with changing elevation gradients, while woody ‘TD’ showed a unimodal pattern, and there was little relationship between woody ‘PD’ and elevation. Herbaceous species exhibited significant phylogenetic clustering at low elevations, where they were influenced by climate, aspect, and tree cover. However, woody species exhibited random patterns across elevations. Herbaceous and woody species’ taxonomic and phylogenetic beta diversity is governed primarily by spatial turnover rather than nestedness. Spatial turnover is caused primarily by EF and DF’s combined influence, but their relative importance differs between herbaceous and woody communities. Therefore, we conclude that the responses of herbaceous and woody plants along elevation gradients in the Helan Mountains are decoupled due to their different adaptation strategies to climate factors in the drylands. These findings are important for understanding the assembly mechanisms driving plant communities in dryland under the context of dramatic increases in drought driven by climate warming.

## Introduction

Mountains are hotspots of biodiversity, covering 24% of the total geographical area and supporting about 50% of the planet’s biodiversity (Körner et al., 2011) They provide refuge for many plants and animals due to the various conditions created by the complex topography and could play an important role in protecting biodiversity (Becker et al., 2007; Körner et al., 2011; Yuancai et al., 2022). Mountains often establish unique local climates along elevation gradients (Wang et al., 2022). Continuous change in landscape and climate factors across elevation leads to variations in resource availability (i.e., light and moisture), creating different microenvironmental conditions within short distances (Becker et al., 2007; Ahmad et al., 2020; Behzad et al., 2022). It leads to a sharp transformation in the ecophysiological adaptation of plants (Jiang et al., 2016) and ultimately alters their diversity patterns along elevation gradients (Boscutti et al., 2018). Because of their steep climatic gradients, mountains are considered sentinels of global warming and, therefore, offer a unique field laboratory for understanding the mechanisms driving the evolution and maintenance of biodiversity along elevation gradients (Körner et al., 2016).

Elevation-diversity relationships have been popular in ecology, biogeography, and biodiversity conservation in recent decades (Körner et al., 2011; Shahriari et al., 2019; Montano-Centellas et al., 2021). Previous studies about biodiversity patterns along elevation gradients found an evident disparity in elevation-richness relationships worldwide (Berhanu et al., 2016; Zhang et al., 2016; Mayor et al., 2017). The maximum species diversity occurs in the mid-elevation range (Grytnes, 2003), but decreases or increases in species richness along elevation have also been documented (Zhu et al., 2009; Berhanu et al., 2016; Qianwen et al., 2022). Yet, contemporary changes in biodiversity across the globe have been investigated based largely on taxonomic diversity (TD; i.e., species identity) (Li et al., 2020), which does not necessarily reflect changes in phylogenetic diversity (PD) (Faith, 1992). To solve this problem, Webb et al. (2002) developed the basis for using phylogenetic data to discover the influence of deterministic processes on community assembly. The method based on PD, considering the phytogeographical affinities that drive community assemblages (Webb et al., 2002), has been used to reveal species diversity in elevation gradients (Dainese et al., 2015; Wang et al., 2022). Combining TD and PD has been increasingly recommended to reveal the underlying driving mechanisms of community assemblages (Li et al., 2020; Macheroum et al., 2021) because it provides more comprehensive information on community assembly from ecological and evolutionary processes at the α and β diversity scales (Li et al., 2020; Du et al., 2021). Approaches that relate local processes to regional and evolutionary processes based on PD or spatial turnover will quantify the elevation-diversity relationship from an ecological and evolutionary perspective (Webb et al., 2002). However, to date, the assembly mechanisms driving plant communities along elevational gradients remain well elusive (Luo et al., 2019).

Theories of community assemblages, including niche-based processes and stochastic processes (Montano-Centellas et al., 2021), considered processes of species dispersal, species persistence, and species coexistence, were used to illustrate the species assembly (Webb et al., 2002; Grigoropoulou et al., 2022). Niche theory highlights the role of environmental filtering (EF; species with specific traits coexist under specific environmental stresses) and biological interactions (competitive exclusion causes limiting similarity) (Wang et al., 2021). Webb et al. (2002) claimed that interspecific competition might result in a community pattern of phylogenetic overdispersion. It might also result in phylogenetic clustering if certain clades have stronger competitiveness than others. Some evidence suggests that high-elevation habitats are generally under harsher conditions (low temperatures, dramatic temperature fluctuations) (Wang et al., 2021), in which plant community compositions are more sensitive to climate change than those of low-elevation habitats (Sproull et al., 2015). In contrast, the neutral theory holds that stochastic fluctuations and dispersal filtering (DF) independently determine the patterns of community assembly (Tilman, 2004; Hubbell, 2005). It is now commonly accepted that community assembly is determined by both processes (Farjalla et al., 2012; Grigoropoulou et al., 2022). Recent studies demonstrate that EF could become more critical for shaping plant community structure in stressful environmental conditions, while DF might dominate under benign conditions (Pouteau et al., 2019). Thus, a shift in the community assembly from overdispersal in low elevations to clustering in high elevations appears to occur. For heterogeneous mountains, however, the species assemblage along elevation gradients may be very complex and show various trends (Jarzyna et al., 2021; Montano-Centellas et al., 2021) for different life-form plants because they have diverged from adaptation strategies to specific environmental factors (Lee and Chun, 2015; Loidi et al., 2021; Macheroum et al., 2021). Moreover, due to different spatial scales and life forms in terms of evolutionary relationships and dispersal ability (Liu et al., 2015; Shahriari et al., 2019), quantifying the relative importance of the EF and DF along elevation gradients remains challenging (Du et al., 2021). Despite knowing that communities are being formed, it remains unclear whether EF or DF dominate in the community assembly of different life forms or how they interact along elevational gradients, especially in arid areas (Macheroum et al., 2021).

Drylands are defined as places with an aridity index of less than 0.65. They encompass over 45% of the planet’s land surface and inhabit over 38% of the Earth’s population (Peng et al., 2020). The drylands of China account for about 10.8% of the world’s drylands, and China is the most drought-affected country in Asia (Huang et al., 2015). The dramatic increase in climate warming-driven drought is considered a potential threat to worldwide biodiversity (Dai, 2010; Jiao et al., 2021). Climate change may aggravate dryland degradation through the alteration of spatial and temporal patterns in temperature, rainfall, solar radiation, and winds, leading to vegetation degradation and ecosystem service loss in the drylands (Macheroum et al., 2021; Gul et al., 2022). Nevertheless, plant diversity patterns and assembly mechanisms in dryland mountain ranges are not well documented. The Helan Mountains, a typical arid and semi-arid region of China with particularly fragile habitats exposed to extreme climate change, are one example (Pang et al., 2013; Cheng et al., 2020). The Helan Mountains are also the boundary line between the grassland and desert areas of Northwest China (Jiang et al., 2007). The vegetation types can be classified as desert grassland, sparse mountain grassland, montane coniferous forest, subalpine scrub meadow or alpine meadow. The vegetation distribution patterns in these regions are strongly influenced by climatic and topographical factors (Cheng et al., 2020; Arif et al., 2022a; Chen et al., 2022). However, to our knowledge, elevation-diversity relationships have not been well revealed in the drylands of China, especially when considering the different life forms of plants.

This study investigated herbaceous and woody communities along elevational gradients in the Helan Mountains of arid and semi-arid regions in Northwest China. Here we integrate taxonomic and phylogenetic diversity and environmental drivers (climatic and topographic variables; Cheng et al., 2020) to elucidate how various ecological mechanisms shape herbaceous and woody community assemblages and to reveal how these communities respond to different environmental drivers along elevational gradients. Specifically, we aimed to answer the following key scientific questions:

(1) How do the TD and PD of herbaceous and woody communities vary with changing elevation gradients in the Helan Mountains?
(2) Which factors in the environment impact the TD and PD of herbaceous and woody communities in this area?
(3) How and to what extent do EF and DF affect the spatial turnover of herbaceous and woody species in this region?

## Materials and methods

### Study area and vegetation surveying

**Figure 1:**
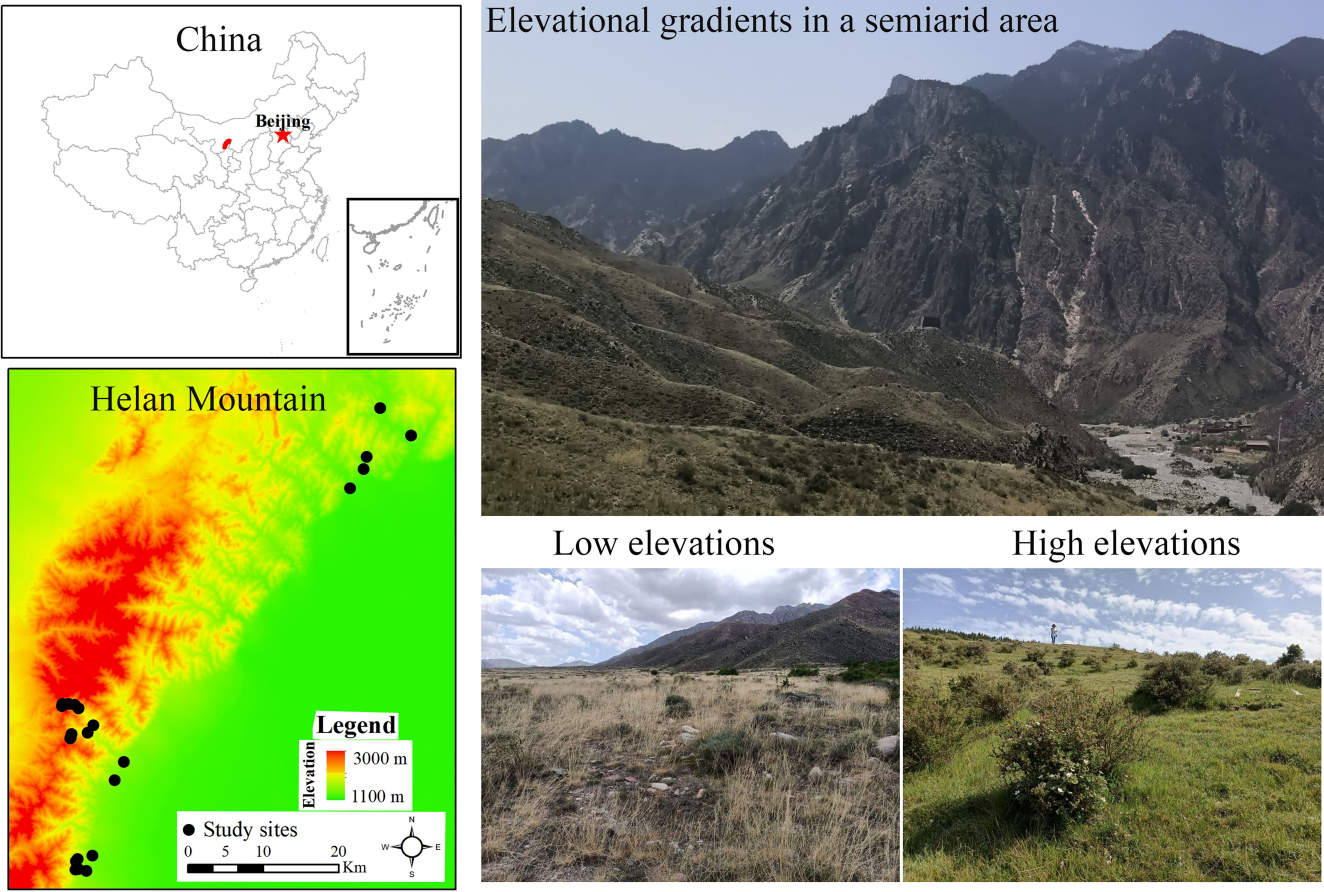
Map of the study site and plots on Helan Mountain of arid and semi-arid regions in Northwest China.

This research was performed in the Helan Mountains (38°13′ N, 105°41′ E; **Figure 1**), which are situated in arid to semi-arid regions in Northwest China. The climate in this region is primarily subject to the influence of the summer and winter monsoons, with mean annual temperatures ranging from 8.2°C to 8.6°C (-8.54°C in January and 21.43°C in August) and mean annual precipitation of 209.2 ± 57.2 mm, of which around 44% of precipitation occurs during the July-August growing season (Pang et al., 2013). High-elevation areas had lower temperatures and higher precipitation than low-elevation areas (Jiang et al., 2007). Gray cinnamonic soils prevail in this area (Wang and Yang, 2021). It is robust evidence of local vegetation evolution in northern China and its relationship with East Asian summer winds (Cheng et al., 2020). Trees (such as *Picea crassifolia*, *Pinus tabuliformis*, and *Ajania fruticulose*), shrubs, and grasses are the main land cover types across this region. However, due to the long lifespan and limited distribution of trees, it is difficult to detect tree distribution patterns with changing elevation gradients. Specifically, an interspersed distribution of continuous woody (shrubs and small trees) and grassland dominates in this area. Considering accessibility, we identified 23 study sites with the peak growing season of 2021, from 1,169 m to 2,589 m in the Helan Mountains. At each site, we established a permanent plot (20 m × 20 m) (Lopez-Angulo et al., 2018), and its vegetation structure and composition were assessed by systematic sampling by using the quadrat method, as it has less bias and covers the entire plant composition (Ahmad et al., 2020). Within each permanent plot, five quadrats of 25 m^2^ (5 m × 5 m for woody) and 1 m^2^ (1 m × 1 m for herbaceous) were sampled (Shi et al., 2020). Each 1 m^2^ quadrat was nested within each 25 m^2^ quadrat. A total of 230 quadrats were sampled. We recorded and measured all the species’ compositions and abundance in each quadrangle. We found 120 species that came from 90 genera and 41 families.

### Taxonomic and phylogenetic diversity metrics

We classified the plants into 44 woody (including all shrubs and trees) and 76 herbaceous species according to the *Flora of China* (http://www.iplant.cn/) and field observations to understand how different species responded to elevational gradients. To compare the TD and PD of herbaceous and woody communities, species-level phylogenetic trees based on the mega-tree approach were constructed using the “V.PhyloMaker” package (Smith and Brown, 2018; Jin and Qian, 2019). The package is the largest dated phylogeny for vascular plants and includes 74,533 species of extant vascular plants (Jin and Qian, 2019). We then used the pruned versions of the tree for calculating the phylogenetic structure of both herbaceous and woody species in each of the 23 communities. We then calculated TD using species richness and PD using the mean pairwise distance (MPD) and the mean nearest taxon distance (MNTD) (Qian et al., 2019). To disentangle the assembly mechanisms of herbaceous and woody communities (Luo et al., 2019), we converted their MPD and MNTD metrics into standardized effect values (SESmpd and SESmntd indices) in the ‘Picante’ package (Kembel et al., 2010). Positive and negative values of SESmpd and SESmntd respectively indicate overdispersal and clustering (Yakimov et al., 2020). Their absolute values are higher than 1.96, meaning that the community structure is significantly overdispersed or clustered compared to null expectations (Webb et al., 2002; Yakimov et al., 2020). In addition, to distinguish TD and PD of herbaceous and woody communities at the beta scale, in the “betapart” package, we calculated three pairwise dissimilarity indices (Baselga, 2010; Baselga and Orme, 2012), in which the Sørensen dissimilarity (βsor) index measures the overall beta diversity, the Simpson dissimilarity (βsim) index measures the turnover component, and the nestedness dissimilarity (βnes) index measures the nestedness component derived from nestedness-related richness differences.

### Environmental and climatic variables

Temperature, precipitation, and their extremes will remarkably influence species’ ecological and evolutionary processes (Qian et al., 2019). Consequently, seven indicators of temperature and precipitation (**Table S1**) from the WorldClim database (Hijmans et al., 2005) were used to represent the climatic characteristics of the study area. Before the analyses, a principal component analysis (PCA; **Table S1**) was used to prevent the effects of collinearity (**Figure S1**) (Dormann et al., 2013), and the PCA axes with an interpretation rate higher than 10% were retained for subsequent analysis (Slik et al., 2013), resulting in two axes of climate variables (PC1 and PC2; **Figure S2**). Besides, the slope and aspect (a proxy for topographic heterogeneity; Shiferaw et al., 2018) of each plot were determined and closely related to the amount of incoming solar radiations and the construction of different microclimatic conditions (Pang et al., 2013), which is a significant factor that promotes diversification in plant community composition. In addition, the altitude, longitude, and latitude of each plot were recorded with GPS.

### Statistical methods

We analyzed the relationship between TD, SESmpd, SESmntd and elevation using simple linear and quadratic regression models (without significant spatial autocorrelation based on Morans’I index, **Table S2**). The analysis considered both linear and quadratic terms but only retained the results for the linear term when the quadratic term was not significant. A linear multiple regression model involving the ordinary least squares (OLS) method was used to identify the role of climate (climate PC1, climate PC2), topographic (slope and aspect), and biotic (woody cover for herbaceous) drivers (**Figure S3**) in determining a regional disparity in the TD and PD of herbaceous and woody species. Prior to the OLS analyses, all predictors were centered and scaled (Chun and Lee, 2017). To understand the relative contribution of predictor variables to the diversity of herbaceous and woody species, model averaging methods based on Akaike information criterion weights were used in the “MuMIn” package (Barton, 2019). We estimated the coefficients by averaging over all possible models and weighting them according to the probability associated with each model.

We investigated the correlation between taxonomic and phylogenetic turnover based on the βsim index with spatial and environmental distance to evaluate the species turnover along altitudinal gradients by employing the “ecodist” package (Goslee and Urban, 2007). A variance partitioning approach was performed to quantify the relative contribution of EF and DF in taxonomic and phylogenetic turnover. Prior to variation partitioning, the redundancy analysis and the forward selection were executed to reduce redundant components (Zheng et al., 2021). In this analysis, the ‘vegan’ package partitions the total explained variance (R^2^) into three parts (Oksanen et al., 2020; Shi et al., 2021), including their unexplained, joint, and independent effects. The Euclidean distances of five environmental variables (PC1, PC2, elevation, aspect, slope) between 23 sites were chosen to reflect EF, and spatial distances between study sites were selected to reflect DF (Li et al., 2021; Shi et al., 2021). We use the adjusted R^2^ to estimate the explanatory power of each component because of the different number of explanatory variables in our models.

In addition, to further examine clustering or overdispersion of plant communities, we performed a co-occurrence analysis by calculating the C-score (Checkerboard score; Stone and Roberts, 1990), which is a widely used index to measure associations between species pairs. We used null model analyses to quantify whether species co-occurrence patterns deviated from the expectations of a random (stochastic) assembly process (Ulrich et al., 2012). The values obtained were standardized to allow comparisons among assemblages using the standardized effect size (SES). The observed C-score was higher than randomized expectations, indicating that pairs of species co-occurred to a lesser extent than expected at random. The magnitude of SES was interpreted as the strength of the effect of deterministic processes on the assemblage. The C-score was evaluated based on 10,000 simulations and using the sequential swap randomization algorithm with the package “EcoSimR” (Gotelli et al., 2015).

## Results

### The TD and PD of herbaceous and woody community

**Figure 2:**
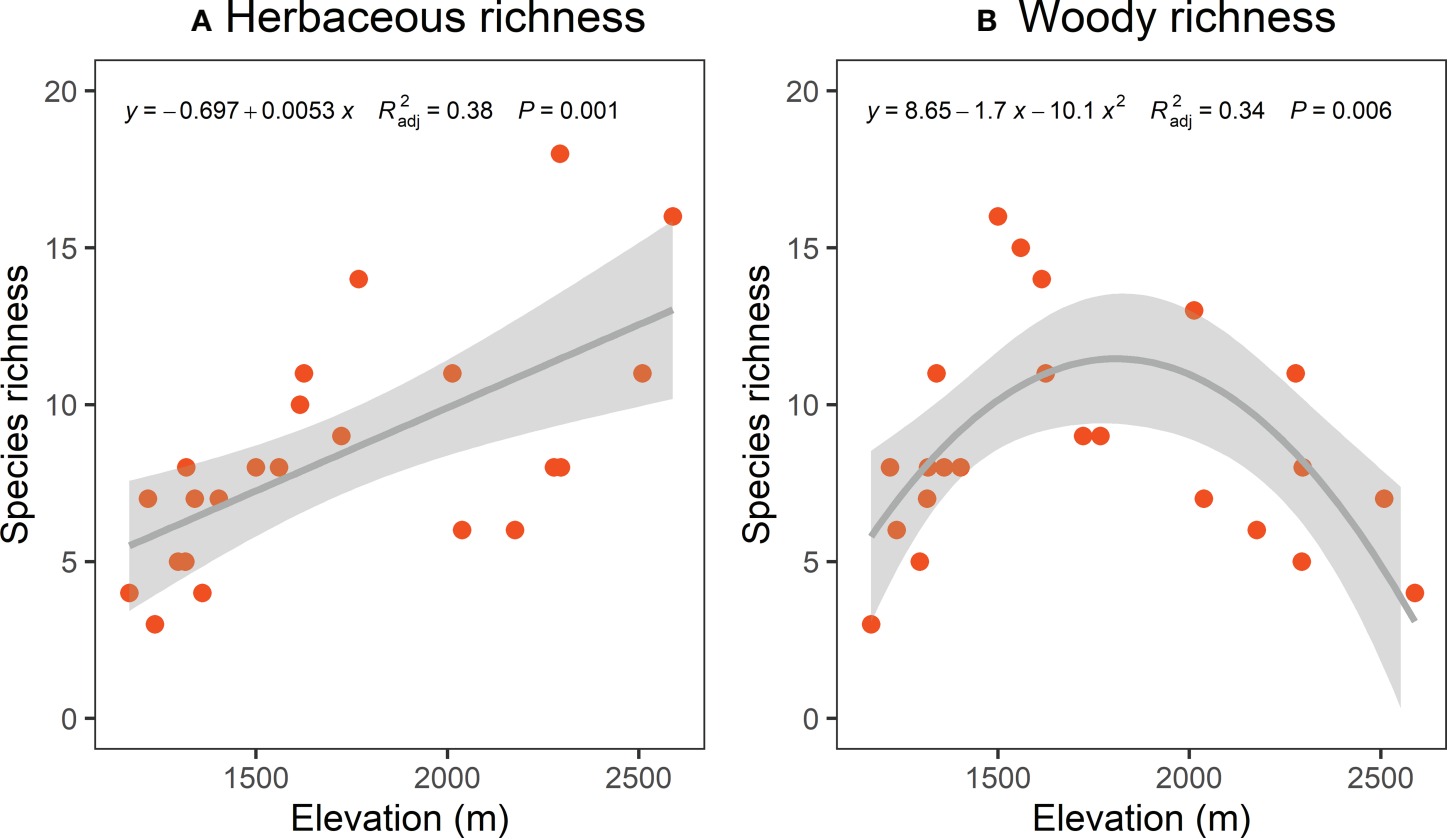
Elevational patterns of species richness of herbaceous **(A)** and woody **(B)**. Trend lines and shaded areas represent the fitted values from linear regression with a linear term **(A)** and quadratic **(B)** and their 95% confidence intervals, respectively.

**Figure 3:**
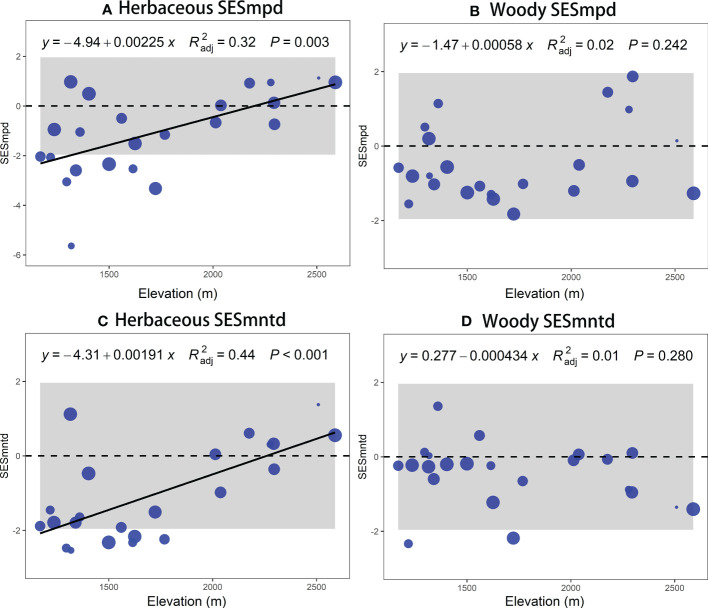
Elevational patterns of standardized values of mean phylogenetic structure (SESmpd and SESmntd) of plant communities of herbaceous **(A, C)** and woody **(B, D)**. The size of the circle indicates the relative species richness associated with each plot.

The TD (species richness) and PD (SESmpd and SESmntd) of herbaceous communities significantly increased linearly along the elevational gradient (**Figure 2A**, **Figures 3A, C** **)**. The nonlinear model was remarkable for woody communities with relatively low richness at the ends of the elevation range and peak richness in the 1500 ~ 1600 m range (**Figure 2B**). However, the PD (SESmpd and SESmntd) of woody communities did not significantly change as elevation increased (**Figures 3B, D**). The SESmpd and SESmntd of herbaceous communities showed random patterns at high elevations (above 2000 m) and were characterized by significant phylogenetic clustering (negative values below ‐1.96) at low elevational sites (**Figures 3A, C**). Surprisingly, all woody communities showed random patterns for SESmpd and SESmntd, except for two communities at low elevations that showed significant phylogenetic clustering for SESmntd (**Figures 3B, D**). Overall, most phylogenetic SESmpd and SESmntd values of herbaceous and woody communities were less than zero, indicating that phylogenetic clustering was driven by deterministic processes. This result was further supported by the co-occurrence analysis, where the observed C-score was higher than the simulated C-score values (**Figure S4**), indicating a non-random co-occurrence pattern. In addition, the C-score showed a higher standardized effect size (SES) for woody plants compared to herbaceous plants, indicating the greater importance of deterministic processes for woody plant assemblages (**Figure S4**).

**Figure 4:**
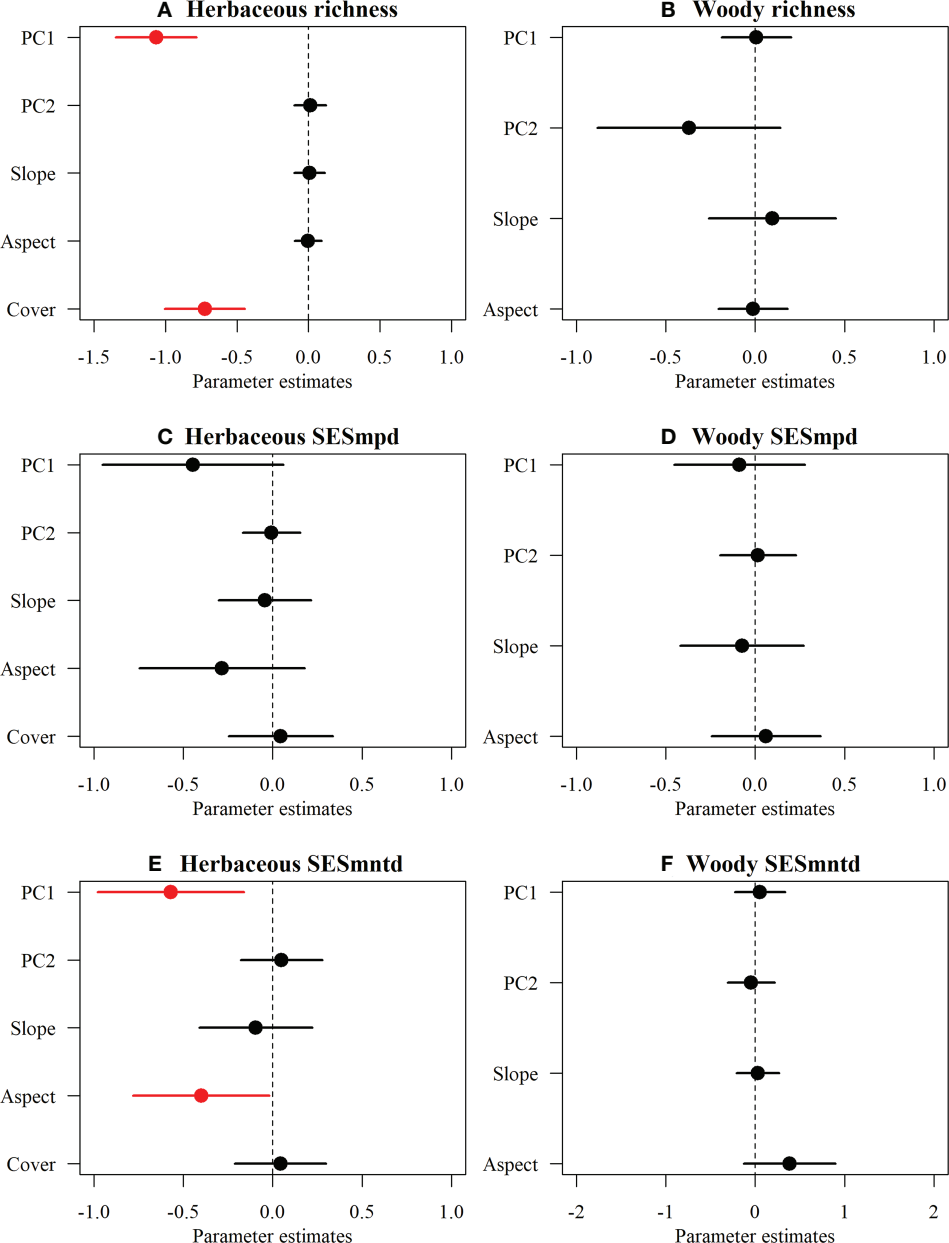
The effects of multi-dimensional variables on the species richness and phylogenetic structure (SESmpd, SESmntd) of plant communities of herbaceous **(A, C, E)** and woody **(B, D, F)**. The parameter estimates (standardized model-averaged coefficients for variables) and the associated 95% confidence intervals are shown. Coefficient > 0 represents a positive effect, while coefficient < 0 indicates a negative effect (red). All predictors were centered and scaled to make the coefficients directly comparable.

For herbaceous communities, climate PC1 and woody cover had significantly negative effects on species richness (**Figure 4A**), whereas SESmntd indices showed significant negative relationships with climate PC1 and aspect, and no relationship between SESmpd and multi-dimensional variables (**Figures 4C, E**). Surprisingly, regional (climatic PC1 and PC2) and local (aspect and slope) variables were not significantly correlated with the TD and PD of woody communities (**Figures 4B, D, F**).

### Taxonomic and phylogenetic beta diversity of herbaceous and woody communities

**Figure 5:**
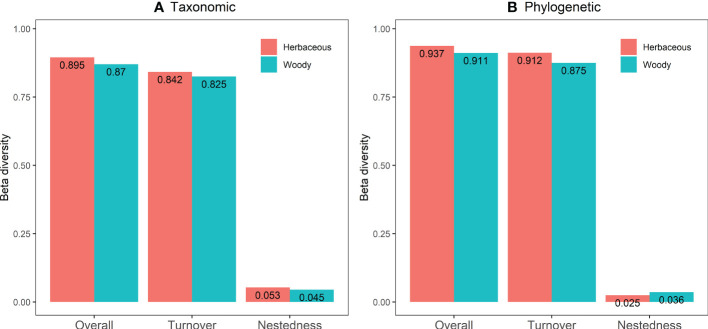
The taxonomic **(A)** and phylogenetic **(B)** β-diversity and its composition of the herbaceous and the woody community in the arid and semiarid areas of China.

Overall variation in the taxonomic and phylogenetic β-diversity of the herbaceous and the woody resulted mainly from turnover rather than nestedness (**Figure 5**). In taxonomic β-diversity, the species turnover of herbaceous and woody were 0.842 (94.08%) and 0.825 (94.83%), whereas species nestedness accounted for only 0.053 (5.92%) and 0.045 (5.17%), respectively (**Figure 5A**). In phylogenetic β-diversity, the species turnover of herbaceous and woody were 0.912 (97.33%) and 0.875 (96.05%), whereas species nestedness accounts for only 0.025 (2.67%) and 0.036 (3.95%), respectively (**Figure 5B**).

For herbaceous communities, the taxonomic turnover was related to environmental distance after accounting for spatial effects (Mantel’s r = 0.42, p = 0.001), but it did not depend on the spatial distances when considering the environmental influences (Mantel’s r = -0.14, p = 0.12). Phylogenetic turnover displayed a similar trend, but it was reduced in prominence when considering spatial influences (Mantel’s r = 0.34, p = 0.001) or environmental effects (Mantel’s r = -0.20, p = 0.06). For woody communities, the taxonomic turnover was related to environmental (Mantel’s r = 0.53, p = 0.001) but it was not associated significantly with spatial distance (Mantel’s r = -0.16, p = 0.06). Phylogenetic turnover was significantly correlated with the environment (Mantel’s r = 0.55, p = 0.001) and spatial distance (Mantel’s r = -2.4, p = 0.011).

**Figure 6:**
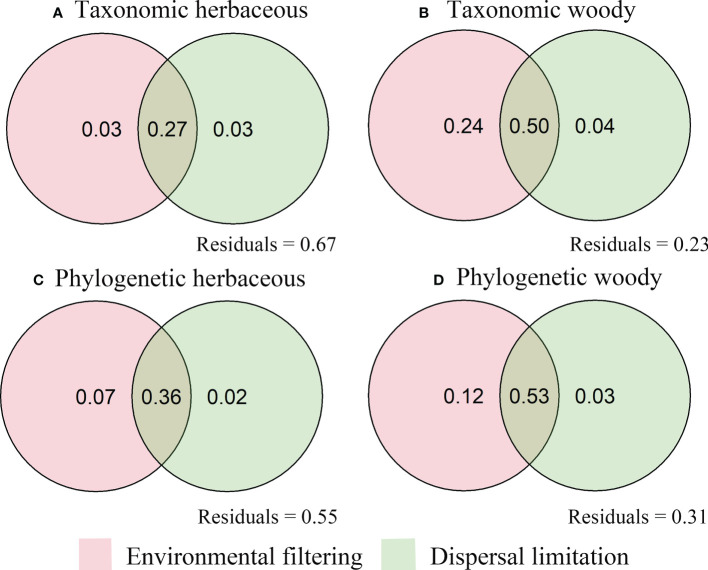
Venn diagram displaying the relative importance of environmental filtering and dispersal filtering on herbaceous **(A, C)** and woody **(B, D)** species turnover in the arid and semiarid areas of China.

Environmental and spatial variables explained 33% to 77% of the variation in species turnover (**Figure 6**). The independent effects of environmental variables accounted for 3% and 7% of the overall variances of taxonomic turnover and phylogenetic turnover, respectively. Independent spatial effects accounted for 3% and 2% of overall variances of taxonomic turnover and phylogenetic turnover (**Figures 6A, C**). In comparison, environmental variables (24% for taxonomic turnover and 12% for phylogenetic turnover) were drastically higher than that spatial variables (4% and 3%) (**Figures 6B, D**). Compared to the pure effects, their combined effects accounted for most of the turnover variation in herbaceous (27% for taxonomic turnover and 36% for phylogenetic turnover) and woody (50% for taxonomic turnover and 53% for phylogenetic turnover) species (**Figure 6**).

## Discussion

### Elevational patterns and drivers of herbaceous and woody in drylands

The species richness of the woody community peaked at mid-elevation areas and declined toward both sides of elevation gradients (**Figure 2B**), supporting the earlier studies in the Helan Mountains (Zhu et al., 2009). The authors attributed the findings to serious drought (low altitude) and lower temperatures (high altitude) with low species richness, and highlighted that the humped pattern of species richness was explained by the moderate climatic situation in the mid-elevations (Zhu et al., 2009). Meanwhile, this result is consistent with previous research on elevational gradients in other mountain habitats (Zhou et al., 2019), which revealed a unimodal relationship. The low richness at lower altitudes might be explained by anthropogenic disturbances and the extreme competition for resources (Sproull et al., 2015; Zhang et al., 2016). On the contrary, the poorer richness at higher altitudes might be explained by the ecophysiological limitations of the harsh environment (Zhu et al., 2009; Luo et al., 2019), in which their growth was hampered and led to poor biodiversity (Ding et al., 2022; Hu et al., 2022). Another possible reason was that abiotic filtration imposed a limit on the seed settlement or species persistence (Montano-Centellas et al., 2021; Wang et al., 2021). In other words, water scarcity reduces species richness at lower altitudes, and low temperatures limit species richness at higher altitudes. In comparison, the greater number of species in mid-elevation regions might be mainly related to favorable climatic conditions (e.g., water availability, optimal temperature) in these regions. This means that pressures are strong at both ends of the elevational gradient, but environmental conditions are milder at mid-elevations and therefore species richness is higher at mid-elevations (Lopez-Angulo et al., 2018).

Unlike woody richness, herbaceous richness showed an increasing tendency along elevational gradients. A study in a subtropical forest supported the pattern that plant species richness increased with elevation (Zhang et al., 2021). They suggest that both human activities and the elevation range described by the study are two key factors leading to the observed diverse patterns (Macheroum et al., 2021; Zhang et al., 2021; Muhammad et al., 2022). In the Helan Mountains, plant communities at high elevations are predominantly herbaceous, whereas those at low elevations are mainly composed of woody plants (Jiang et al., 2007; Pang et al., 2013). The study reported that environmental conditions were generally more beneficial for alpine plants, which seem to have essential roles in herbaceous plants (Yakimov et al., 2020). When compared to species at lower elevations, alpine meadow plants without woody constraints use different survival strategies in harsh environments, such as investing more resources in reproductive and below-ground components and expanding display areas to attract pollinators (Wang et al., 2021). Therefore, the shorter life cycle of herbaceous plants and the higher potential for seed dispersal and germination strategies that favor survival may be the main contributors to changes in herbaceous richness across elevational gradients in the Helan Mountains. Furthermore, our findings demonstrate the combined effect of biotic and abiotic stresses on herbaceous species distribution and support the inconsistent trends of herbaceous and woody richness along altitudinal gradients. Notably, our results appear contrary to the widely reported view that elevational trends of species richness should decrease in a unimodal or monotonic pattern (Lee and Chun, 2015; Sproull et al., 2015; Zhou et al., 2019). However, when drought is exacerbated at lower elevations, it might reduce species diversity and likely produce unexpected biodiversity patterns (Lopez-Angulo et al., 2018). In addition, given the short lifespan of herbaceous plants, especially annual herbs, we may have overlooked species coexistence patterns associated with them due to the fact that we used only one field survey. Therefore, such limitations should be taken into account when interpreting our results. Huang et al. (2016) emphasize that it is essential to pay more attention to herbaceous plants in biodiversity conservation. So, we suggested that more research be done to discover their general distribution in the dryland mountains.

In addition, this study found that both the SESmpd and SESmntd of herbaceous communities significantly increased linearly along the elevational gradient. Still, there is no clear pattern in the phylogenetic structure of woody communities. This difference can be traced to disparities in the responses of various plant functional groups toward climate change, topographic heterogeneity, soil nutrients, and disturbances along the altitudinal gradient in mountainous areas (Paz et al., 2021). In the Helan Mountains, elevation, local conditions, slope, and aspect were reported to strongly influence spatial patterns of plant biodiversity (Jiang et al., 2007; Pang et al., 2013). Compared to woody species, herbaceous plants were significantly constrained by multi-dimensional variables (e.g., climatic, aspect, and woody cover), indicating that the effects of various environmental factors on different groups of life forms were inconsistent (Loidi et al., 2021). Such variations probably relate to differences in longevity and tolerance to weather (Qian et al., 2014). Large differences in life cycles and tolerance to climatic stress between herbaceous and woody plants may result in inconsistencies in diversity distribution patterns across elevations (Qian et al., 2014). Herbaceous plants are generally able to adjust to novel climate situations two to ten times quicker compared to woody plants due to their shorter reproductive cycles and faster growth rates (Smith and Beaulieu, 2009; Zhou et al., 2019). Moreover, their seed germination, dispersal, and establishment may be tightly controlled by climate and other environmental factors (Wang et al., 2021). In our study, after controlling for other variables, herbaceous richness showed a negative correlation with woody cover, which could be that woody species act as a limiting factor in the species composition of herbaceous communities through the reduction of available resources such as light and soil (Sproull et al., 2015). Therefore, herbaceous and woody assembly processes may be driven by different mechanisms (Luo et al., 2019). We suggest that the herbaceous communities are radically distinct from woody communities concerning the phylogenetic structure (Qian et al., 2014), mainly attributed to abiotic and biotic factors (Gao et al., 2017). In mountainous areas that have been grazed and logged for a long time, different results may be found (Dainese et al., 2015; Wang et al., 2019). Therefore, more field experiments in the mountain region are required to validate the above hypothesis.

Although the taxonomic and phylogenetic patterns of elevation distribution of herbaceous and woody plants were not consistent, our study indicated (**Figure 3**) that most herbaceous and woody communities showed phylogenetic clustering (both SESmpd and SESmntd < 0) throughout elevation gradients. Another important finding was that the phylogenetic structure of most communities did not differ significantly from that expected from the expected null model. We believe that this phylogenetic pattern might stem from deterministic and stochastic processes. Environmental filters played a big role in the phylogenetic clustering of SESmpd, which had a big impact on the whole lineage (Zobel and Scheiner, 2016). However, the SESmntd might result from the decentralization capability of contemporary species (Volis and Bohrer, 2013). In the Helan Mountains, drought has likely filtered out species that lack drought tolerance mechanisms. Preliminary research has shown that strong environmental filtering procedures are more likely to form community assemblages at high altitudes than competitive exclusion procedures (Chun and Lee, 2017). Other studies support that herbaceous species may be subject to assembly processes (Luo et al., 2019), such as dispersal limitation due to their smaller body and fruit size (Jiang et al., 2015). Moreover, the stochasticity may promote the early assembly of plant communities due to the limitations of seeds (Farjalla et al., 2012). Apparently, niche-based and random processes may play a big role in how plants are put together in the Helan Mountains.

### Ecological drivers of species turnover

Here we found a comparatively high taxonomic and phylogenetic species turnover of herbaceous and woody communities in the Helan Mountains flora (**Figure 5**), which is consistent with research in Donglingshan Mountain (Yakimov et al., 2020). Although herbaceous and woody plants exhibit a higher taxonomic and phylogenetic turnover, they correlated poorly with spatial distance after controlling for environmental effects. As previous studies have shown, an environmentally heterogeneous ecosystem generally has a relatively high turnover rate (Coelho et al., 2018). Species turnovers tend to increase with environmental heterogeneity due to amplified variations in community composition and species richness (Ahmad et al., 2020). There is an evident species substitution phenomenon in this highly heterogeneous mountain ecosystem due to regional environmental differences (Bergamin et al., 2017). In addition, turnover is related to elevation and may also be influenced by soil fertility, water resources, meteorology, and light conditions (Dainese et al., 2015; Nascimbene and Spitale, 2017).

This study found that independent contributions of spatial and environmental factors differentially influenced taxonomic and phylogenetic turnovers of herbaceous and woody species (**Figure 6**). The reason could be that herbaceous and woody plants differ in their evolutionary relationships and dispersion patterns (Liu et al., 2015). Studies show that woody plants are both less plastic in their basic niche and more dispersal-selected than herbaceous plants (Farjalla et al., 2012). Our study area’s topographic structure may have resulted in significant geographic isolation, limiting the migration, dispersal, and assemblage of many of the species distributed in the current forest. Further, canopy structure might more intensely influence understory plant (herbaceous) composition (Luo et al., 2019). Consequently, they probably followed relatively independent pathways in terms of evolution, dispersal filtering, and species interactions. We also found that the joint contribution of spatial and environmental factors had the highest proportion in explaining the turnover of herbaceous and woody plants (**Figure 6**). This result means that the spatial turnover of plant communities in our study area is mainly shaped by a combination of EF and DF (Chun and Lee, 2017; Grigoropoulou et al., 2022). As emphasized by previous studies, our results support the view that EF and DF jointly control plant community assembly, although the relative contribution of these two processes at different areas and scales is still inconclusive (Shi et al., 2021). It is worth noting that herbaceous and woody species are constantly interacting. Woody species can act as a filter in the structuring of understory plants (herbaceous) by reducing resource availability (Luo et al., 2019), but the sampling design of this study did not proceed according to plant-plant interactions, which would not directly capture the effect of woody plants on herbaceous plants. Nevertheless, integrating the taxonomic and phylogenetic diversity of herbaceous and woody species might provide a novel insight into the flora assemblage in the arid and semi-arid mountains.

### Implications of biodiversity conservation

As a crucial dimension of biodiversity, beta diversity could offer important evidence to predict ecosystem functioning and improve protected priorities (Martinez-Almoyna et al., 2019; Scriven et al., 2015). Furthermore, knowledge of beta diversity patterns may effectively shape preservation policies (Swenson, 2011; Bergamin et al., 2017). For example, if species turnover is the dominant pattern, more protected areas are needed for biodiversity conservation (Arif et al., 2022b; Hira et al., 2022). However, when nestedness is the prevailing pattern, there is a need to establish a sufficiently large protected area with high species richness (Bergamin et al., 2017). Given that species turnover in the Helan Mountains is the dominant factor in driving the β-diversity pattern (**Figure 5**), conservations must focus on a vast number of protected areas distributed to provide maximum protection for the life forms of species (Bergamin et al., 2017; Muhammad and Changxiao, 2022). Increasing evidence suggests that phylogenetic diversity is a key to understanding species assemblages and might lead to a fundamental understanding of ecosystem function (Li et al., 2020). Phylogenetic diversity represents the lineage evolution relationships among species and it can help form conservation strategies, it may be more important to conserve a phylogenetically unique species, compared to a more redundant one. Furthermore, our results suggest that both environmental and spatial factors play an important role in shaping plant community assembly, and therefore, it is desirable to combine environmental filtering with dispersal limitation in the practical process of biodiversity conservation in drylands. As a consequence of climate change, the extent of global dryland area is projected to increase particularly in developing countries, which further increases dryland degradation and biodiversity loss (Huang et al., 2015). The global decline in biodiversity has driven calls for ambitious targets for biodiversity conservation and protected areas coverage (Peng et al., 2021). Thus, our findings could have a big impact on the conservation of biodiversity in drylands as the climate warms and causes more drought (Zhu et al., 2020; Jiao et al., 2021).

## Conclusions

In summary, this study integrates TD, PD, and environmental drivers to evaluate herbaceous and woody species’ general patterns and assembly mechanisms along elevation gradients in arid and semi-arid regions. Our study demonstrated that the responses of herbaceous and woody plants along elevation gradients in the Helan Mountains are decoupled mostly due to the inconsistent influence of biotics and abiotics on them. The TD and PD of herbaceous communities substantially increase linearly along an elevational gradient, while a nonlinear model was remarkable for woody communities with relatively low richness at the ends of the elevation range and peaked richness at mid-altitudes. In addition, there was no relationship between woody PD and elevation. We found TD and PD were mainly dominated by species turnover with fewer contributions from nestedness. The turnover process is primarily caused by a combination of environmental and spatial variables. We also showed that environmental and dispersal filtering jointly shapes the plant community assemblages along elevational gradients in arid and semi-arid areas. These findings highlight that conservationists and policymakers should focus on the different adaptation strategies of herbaceous and woody plants to the drought that continues to increase with global warming.

## Data availability statement

The original contributions presented in the study are included in the article/**Supplementary Material**. Further inquiries can be directed to the corresponding author.

## Author contributions

Conceptualization, Methodology, Software, Validation, Formal analysis, Investigation, Resources, Data curation, Visualization, Writing - original draft, Writing - review, and editing were performed by JZ. Methodology, Software, Validation, Formal analysis, Investigation, Writing - review, and editing was performed by MA. Investigation, Writing - review, and editing were performed by XH and DD. Validation, Writing - review, and editing were performed by SZ. Investigation, Formal analysis, Data curation was performed by XN. Investigation, Resources, Writing - review and editing, Supervision, Project administration, Funding acquisition were performed by CL. All authors contributed to the article and approved the submitted version.

## Funding

This work was supported by Ningxia Key Research and Development Project (No. 2020BFG03006, 2021BEG02005); Ningxia Natural Science Foundation Project (No. 2020AAC03107); Chongqing Municipality Key Forestry Research Project (No. 2021-9); Chongqing Municipality Housing and Urban Construction Committee (No. Chengkezi 2019-1-4-2); Forestry Extension Project of China Central Finance (No. Yulinketui 2020-2); Science Foundation of College of Life Sciences of Southwest University (No. 20212005406201).

## Acknowledgments

We are grateful to Mr. Zhu Qiang and Mr. Wang Jifei for their technical and field support during data collection. Finally, we want to express our profound gratitude to Mr. Yan Lingbing for his immeasurable support.

## Conflict of interest

The authors declare that the research was conducted in the absence of any commercial or financial relationships that could be construed as a potential conflict of interest.

## Publisher’s note

All claims expressed in this article are solely those of the authors and do not necessarily represent those of their affiliated organizations, or those of the publisher, the editors and the reviewers. Any product that may be evaluated in this article, or claim that may be made by its manufacturer, is not guaranteed or endorsed by the publisher.
