# Supplementary material for: Plant community assembly is jointly shaped by environmental and dispersal filtering along elevation gradients in a semiarid area, China
Source: Front Plant Sci. 2022 Nov 25;13:1041742. doi: 10.3389/fpls.2022.1041742 (PMC9732563; doi:10.3389/fpls.2022.1041742)
Supplement: Supplementary file 1 [file DataSheet_1.docx]

Supplementary Material

## Supplementary Figures


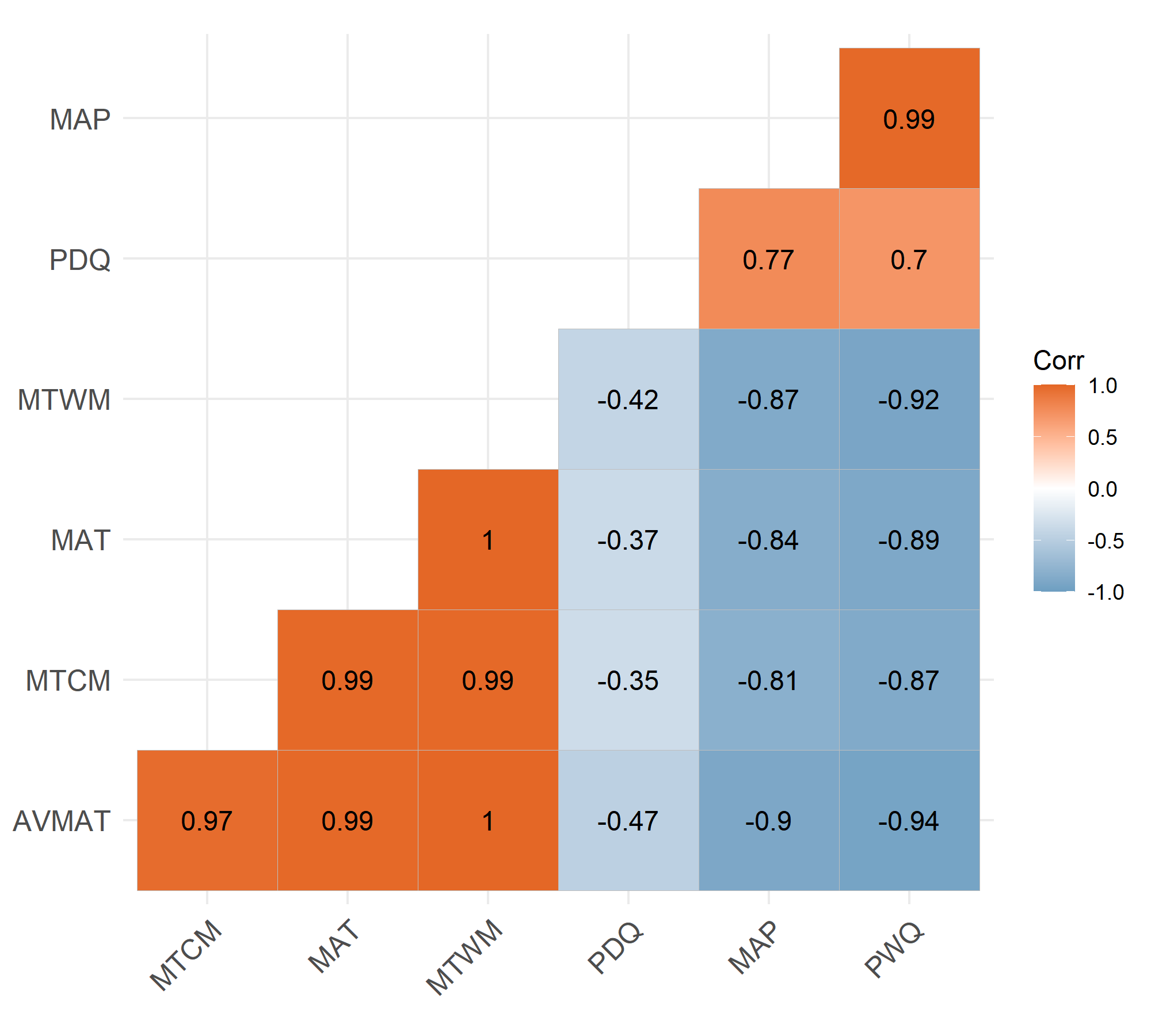


**Supplementary Figure 1.** Pairwise Pearson’s correlation between climatic variables. MTCM = mean temperature of the coldest month; MAT = mean annual temperature; MTWM = mean temperature of the warmest month; PDQ = precipitation of driest quarter; MAP = mean annual precipitation; PWQ = precipitation of wettest quarter; AVMAT = mean annual temperature.


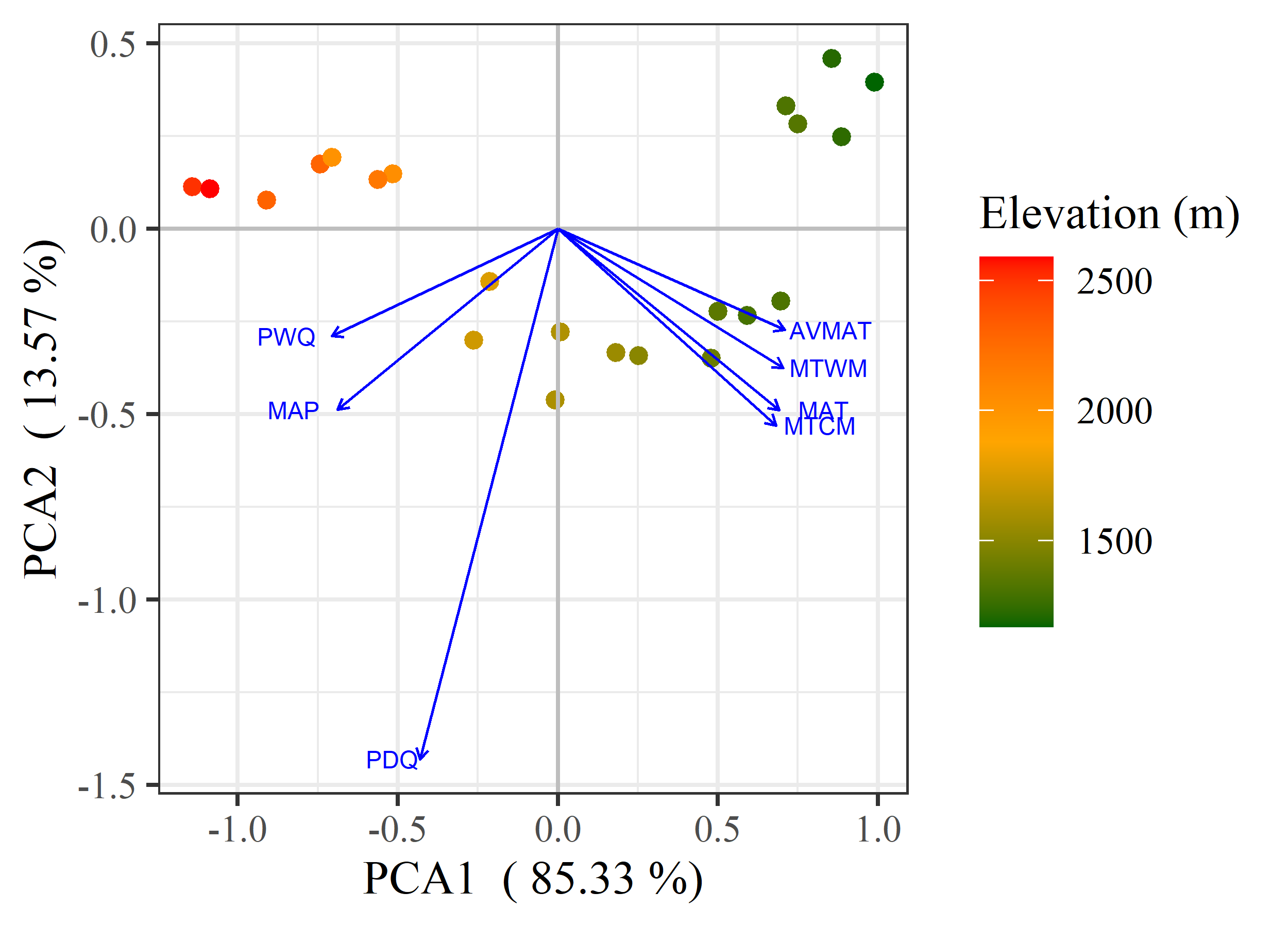


**Supplementary Figure 2.** The principal component analysis (PCA) was performed for climatic variables. Climate variables (climate PC1, climate PC2: the scores of the first two axes of climate variables). MTCM = mean temperature of the coldest month; MAT = mean annual temperature; MTWM = mean temperature of the warmest month; PDQ = precipitation of driest quarter; MAP = mean annual precipitation; PWQ = precipitation of wettest quarter; AVMAT = mean annual temperature.


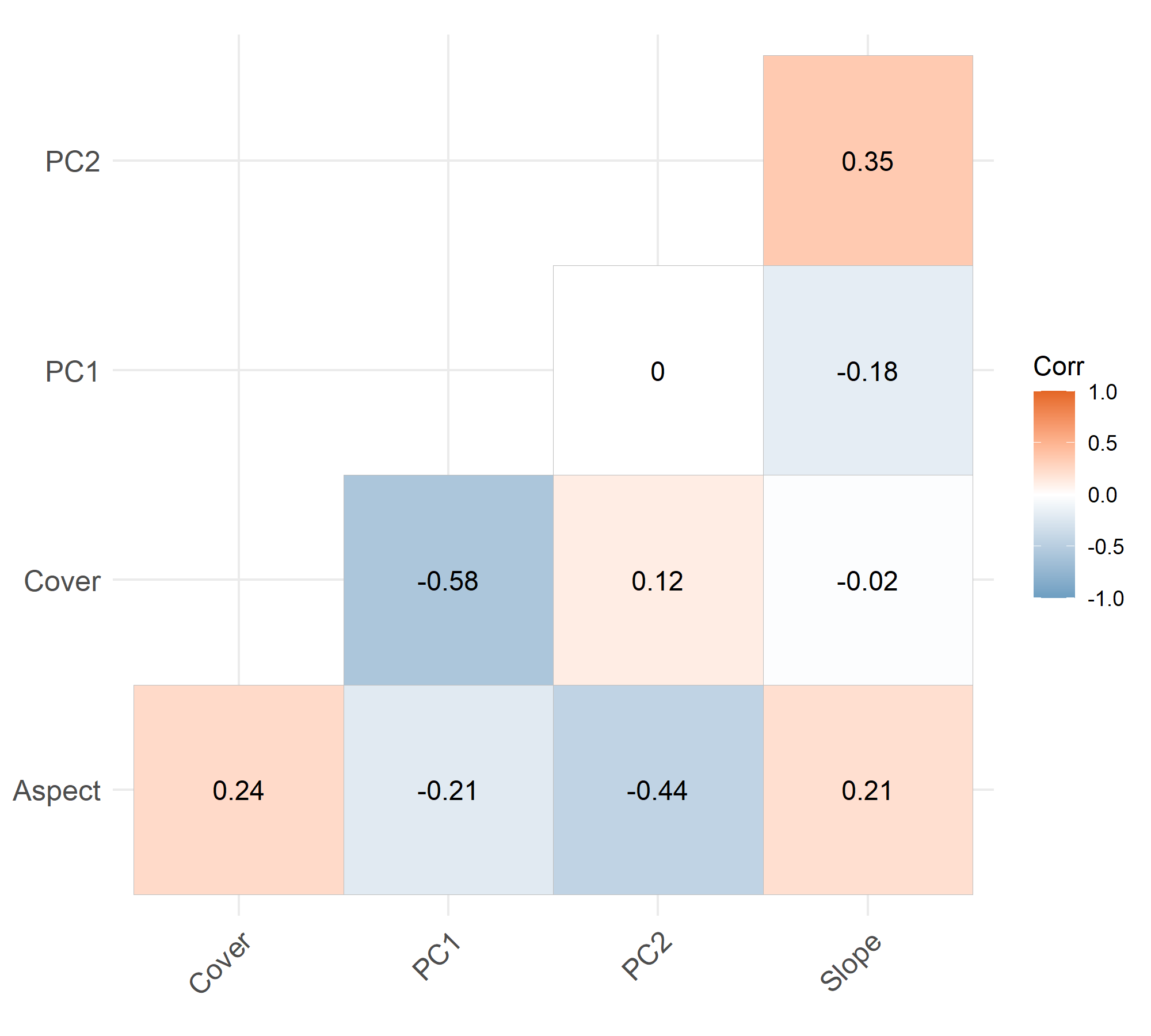


**Supplementary Figure 3.** Pairwise Pearson’s correlation between climate variables (climate PC1, climate PC2), topographical variables (slope, aspect), and woody cover.


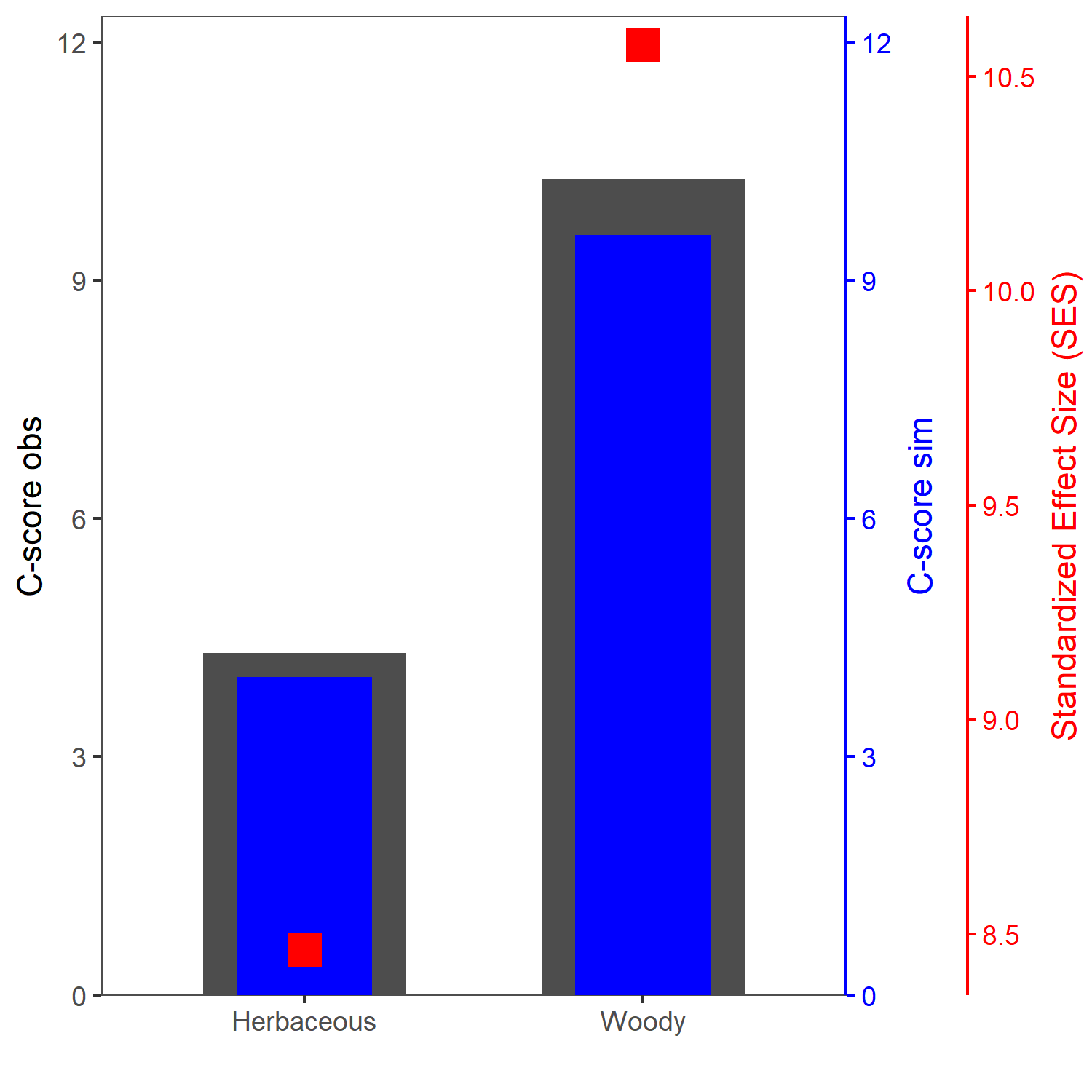


**Supplementary Figure 4.** The results of co-occurrence analysis for herbaceous and woody plants using null models. The values of observed C-score (C-score obs) > simulated C-score (C-score sim) indicate non-random co-occurrence patterns. Standardized effect size <− 2 and > 2 represent aggregation and segregation, respectively.

## Supplementary Tables

**Supplementary Table 1** Seven variables about temperature and precipitation and the results of their principal component analysis. MTCM = mean temperature of the coldest month; MAT = mean annual temperature; MTWM = mean temperature of the warmest month; PDQ = precipitation of driest quarter; MAP = mean annual precipitation; PWQ = precipitation of wettest quarter; AVMAT = mean annual temperature

|  | Climatic variables | PC1 | PC2 | PC3 | PC4 |
| --- | --- | --- | --- | --- | --- |
| 1 | MAP | -1.376 | -0.975 | -1.784 | -0.586 |
| 2 | PDQ | -0.863 | **-2.862** | 1.814 | 0.377 |
| 3 | PWQ | **-1.412** | -0.579 | -1.669 | -0.998 |
| 4 | MAT | 1.384 | -0.977 | -0.762 | -0.634 |
| 5 | MTCM | 1.365 | -1.061 | -1.522 | 2.225 |
| 6 | MTWM | 1.409 | -0.748 | -0.395 | -0.565 |
| 7 | AVMAT | **1.419** | -0.545 | 0.311 | -2.293 |
|  | Eigenvalues | 5.973 | 0.950 | 0.057 | 0.017 |
|  | Percentage | 0.853 | 0.136 | 0.008 | 0.002 |
|  | Cum. Percentage | 0.853 | 0.989 | 0.997 | 1.000 |

**Supplementary Table 2** Moran’s I coefficient for the model's studentized residuals on species richness, the standardized effect size of the mean pairwise distance (SESmpd) and mean nearest taxon distance (SESmntd) in both herbaceous and woody plant communities along the elevation gradient

| Community | Variable | Observed | Expected | SD | p-value |
| --- | --- | --- | --- | --- | --- |
| Herbaceous | Species richness | -0.219 | -0.045 | 0.107 | 0.105 |
|  | SESmpd | -0.021 | -0.045 | 0.103 | 0.815 |
|  | SESmntd | 0.121 | -0.045 | 0.099 | 0.092 |
| Woody | Species richness | 0.130 | -0.045 | 0.107 | 0.102 |
|  | SESmpd | -0.118 | -0.045 | 0.108 | 0.505 |
|  | SESmntd | -0.031 | -0.045 | 0.103 | 0.890 |
